# Interface-Engineered Sodium Alginate-Based Fire-Suppressing Gel: Strong Rheology and Efficient Gas–Solid Flame Retardancy via N-P Coupling

**DOI:** 10.3390/gels12050363

**Published:** 2026-04-27

**Authors:** Xiaoxu Gao, Haiyang Wang, Haochen Li, Jie Yang, Xuetao Cao

**Affiliations:** 1 School of Energy Engineering, Xi’an University of Science and Technology, Xi’an 710054, China; 18729365702@163.com; 2 Key Laboratory of the Ministry of Education on Mining and Disaster Prevention in Western China, Xi’an 710054, China; 3 School of Safety Science and Engineering, Xi’an University of Science and Technology, Xi’an 710054, China; 4 College of Energy and Mining Engineering, Shandong University of Science and Technology, Qingdao 266590, China

**Keywords:** nitrogen–phosphorus coupling, sodium alginate, rheology, bio-based gel, SiO_2_-APTES interface

## Abstract

Environmental fires pose a serious threat to energy security, ecosystems and public safety, whilst traditional halogenated flame retardants suffer from limitations such as high environmental residue risks and insufficient flame-retardant efficacy. In this study, sodium alginate (SA) was utilised as the matrix, with the incorporation of ammonium polyphosphate (APP) and phytic acid (PA), in conjunction with SiO_2_-APTES surface modification, to prepare nitrogen–phosphorus synergistic bio-based flame-retardant gels. The present study systematically investigated the influence of the N/P molar ratio on the gelation kinetics, rheological behaviour, microstructure and flame-retardant performance of the gel. The study revealed a nitrogen–phosphorus coupled gas–solid two-phase synergistic flame-retardant mechanism. The results indicate that at an N/P ratio of 1/4, the gel forms a stable dual-network structure comprising ionic cross-links and Si–O–P covalent bonds. In the gas phase, the thermal decomposition of APP releases inert NH_3_, which dilutes oxygen and quenches gas-phase radicals (·OH, ·H). In the condensed phase, the phosphate groups of PA-catalysed SA form Si–O–P covalent bonds with SiO_2_ under the mediation of APTES, creating a dense, insulating char layer. In comparison with the control group (N/P = 0/0), the optimal gel sample (N/P = 1/4) demonstrated a 33% increase in shear stress, a 10% reduction in the peak heat release rate (HRR), a 75% decrease in total smoke production (TSP), and a 150% increase in char layer thickness after combustion, while maintaining adequate mechanical strength, thermal stability, and environmental friendliness. This work provides novel insights and strategies for the development of green, highly efficient flame-retardant materials for environmental fire prevention and control.

## 1. Introduction

Global coal mine and forest fires pose severe threats to energy security and ecology [1,2]. Coal fires are a particular problem, resulting in substantial coal resource waste and the release of large quantities of greenhouse gases and toxic fumes. They severely disrupt the ecological equilibrium of mining areas and frequently trigger secondary disasters, such as gas explosions [3]. Statistics indicate that hundreds of millions of tonnes of coal are lost globally each year due to coal fires, with management and restoration costs continuously escalating [4]. Concurrently, under the SSP370 and SSP585 scenarios based on IPCC AR6 climate projections, forest fire risks are projected to increase by 30.6% and 35.4% respectively, with smoke-induced premature deaths potentially reaching 1.4 million annually [5,6]. Concerns over the environmental persistence of traditional halogenated flame retardants are growing. There is therefore an urgent need to develop novel fire-extinguishing materials that combine high flame retardancy with environmental compatibility [7,8,9,10].

Currently, coal mine and forest fires are primarily prevented and controlled using water-based fire retardants, chemical foams, and gel fire suppression systems. However, these technologies have significant limitations in complex fire scenarios. Although water-based materials are inexpensive and environmentally friendly, they evaporate easily and do not adhere well, making them ineffective against deep-seated coal fires or smouldering sources [11,12]. Although fluorinated foams offer strong adhesion, they are difficult to degrade and can contribute to persistent organic pollution [13]. Although some studies have improved the efficacy of aqueous film-forming foams for fire suppression by optimising spray pressure and mixing ratios, this has not resolved the fundamental conflict between environmental sustainability and long-term efficacy.

Due to their renewability, high water retention capacity and excellent environmental compatibility, bio-based gel materials have gradually emerged as a research focus in the field of coal mine and forest fire prevention and control [14,15]. These materials form dense protective layers rapidly at elevated temperatures, achieving flame retardancy through mechanisms such as oxygen isolation, adsorption of combustible gases, and heat absorption-induced cooling. They demonstrate significant potential in preventing and suppressing coal mine goaf fires, storing flammable materials, and rescuing people in forest fires [16,17]. Sodium alginate (SA), a natural polysaccharide polymer, rapidly forms highly water-retaining gel networks through cross-linking with ions such as Ca^2+^, effectively coating coal or forest surfaces [18,19]. Although relevant studies have reported the synergistic flame-retardant effects of nitrogen and phosphorus [20,21,22,23], existing work has largely focused on the performance of combustion suppression. This has not only overlooked the critical regulatory role of the N/P molar ratio in gelation kinetics, three-dimensional network formation, and rheological properties, but has also failed to systematically investigate the gas–solid two-phase flame-retardant behaviour of SA-SiO_2_ composite gels. Furthermore, issues regarding the structural stability and dispersion of nanoparticles under high-temperature conditions remain unresolved. While studies have confirmed that nitrogen–phosphorus modification can enhance the thermal stability of materials, research has largely focused on the effects of individual flame-retardant elements, with little systematic analysis of the synergistic mechanisms of nitrogen and phosphorus in bio-based gel systems. In particular, the mechanisms by which the N/P molar ratio regulates the gel network microstructure, rheological properties and gas–solid two-phase flame retardant behaviour remain unclear. Moreover, the structural stability and long-term coverage capability of existing gel systems at high temperatures must be improved; this is a key issue limiting their practical application in preventing and controlling the entire coal spontaneous combustion process [24].

Based on this, the present study used sodium alginate as the matrix [25,26], incorporating APP and PA to create a nitrogen–phosphorus flame retardant system. SiO_2_-APTES interface modification techniques were employed using silica sol (SiO_2_) nanoparticles and a silane coupling agent (APTES) [27] to prepare a high-performance, environmentally friendly, bio-based gel suitable for extinguishing and preventing coal spontaneous combustion as well as forest surface fires via pouring or spreading methods. The N/P molar ratio is systematically optimized to regulate gelation, rheology, microstructure, and flame retardancy. The gas–solid biphasic mechanism and Si-O-P interfacial interaction are revealed, providing a green gel strategy for fire prevention.

## 2. Results and Discussion

### 2.1. Characterization of Basic Parameters of Bio-Based Gel

#### 2.1.1. Gelling Time Analysis

**Figure 1:**
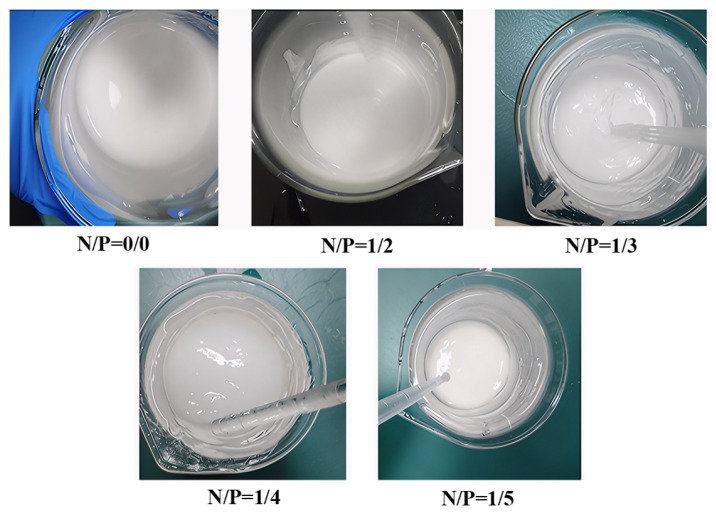
Flow chart of substrate preparation.

Gelation time is a pivotal parameter in the assessment of gel materials’ practical applicability, directly impacting their operability and application efficiency at forest fire sites [28,29]. The gelation time of gels with different N/P molar ratios (0/0, 1/2, 1/3, 1/4, 1/5) was determined using the bottle test method [30]. Three parallel experiments were conducted to select the optimal result, as illustrated in Figure 1. The results indicate that the N/P = 1/3 group had the shortest gelation time, averaging about 3 min, and was able to form a uniform and dense solid network structure; the N/P = 1/2 and N/P = 1/4 groups had a gelation time of about 5 min and could also form solid gels; whereas the control groups (N/P = 0/0, 1/5) were unable to gel, showing a sudden drop in shear stress, which is directly related to the chemical bonding strength

The incapacity of the control group to form a gel can be ascribed to the absence of NH_4_^+^ ions dissociated from APP within the system. In the absence of APP, the carboxylate anions (−COO^−^) on the sodium alginate molecular chains adopt a highly extended conformation, a consequence of electrostatic repulsion between like charges. The connection of these anions with the silica sol nanoparticles is solely reliant on secondary physical interactions, such as hydrogen bonding. This weak cross-linking is insufficient to construct a stable three-dimensional network structure [31]. Following the introduction of an appropriate amount of APP, the NH_4_^+^ ions released act as counterions to form electrostatic complexes with the −COO^−^ groups. The charge-shielding effect is pivotal in neutralising the net negative charge of the molecular chains, thereby facilitating interchain approach and entanglement, and promoting the formation of the gel framework.

However, when the molar ratio of nitrogen to phosphorus (N/P) in the system deviates from the optimal range, significant differences in the gelation behaviour are observed. At an N/P ratio of 1/5, due to an excess of the phosphorus source, phytic acid, the pH of the system drops sharply to approximately 1.0. It has been demonstrated that this strongly acidic environment induces the carboxylate groups of sodium alginate to undergo protonation, thereby converting them into the electrically neutral carboxylic acid form (−COOH). This process has been shown to inhibit NH_4_^+^-mediated ionic cross-linking. Furthermore, the excess phytic acid molecules, due to their significant steric hindrance, physically impeded the effective approach and entanglement of sodium alginate chains, thus preventing the formation of a three-dimensional gel network. Conversely, the system with an N/P ratio of 1/3 demonstrated optimal gelation performance. In such circumstances, the NH_4_^+^ concentration and the ratio of phosphate groups derived from phytic acid to phosphate ester groups derived from phytic acid are measured. It has been demonstrated that, on the one hand, this provides sufficient ionic cross-linking sites to promote the cross-linking of sodium alginate chains; on the other hand, it effectively anchors the silica sol nanoparticles through the multidentate coordination ability of phytic acid. The synergy of these two factors constructs a stable and uniform organic-inorganic composite cross-linking network, thereby significantly shortening the gelation induction time.

#### 2.1.2. Analysis of Viscosity and PH Value

**Figure 2:**
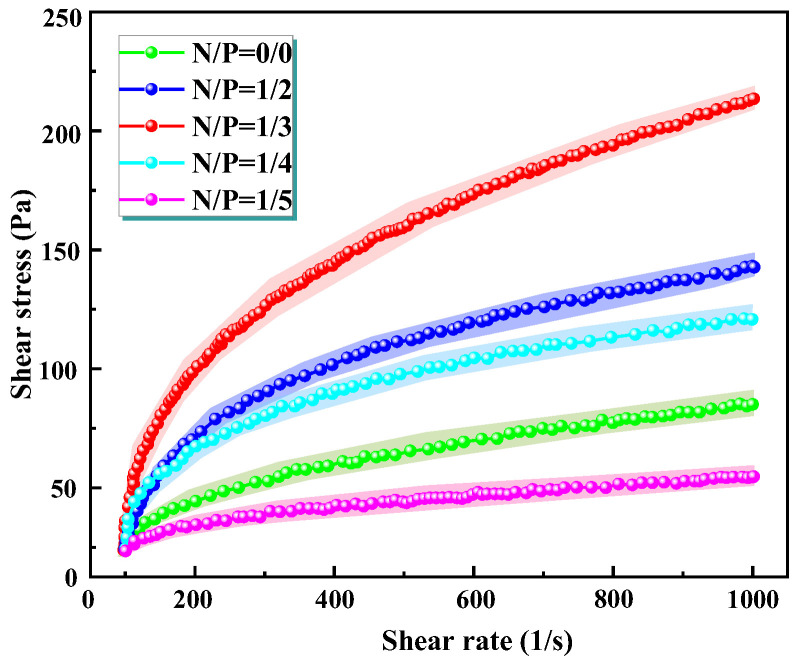
Shows the apparent viscosity curve of the gel with N/P of 0/0, 1/2, 1/3, 1/4, and 1/5 molar mass ratios.

The rheological behaviour of gels is a crucial indicator of their mechanical properties and engineering applicability. The present study utilised a shear rheometer to ascertain the variation in shear stress with shear rate for gels exhibiting divergent N/P ratios. The apparent viscosity of the gel materials was calculated based on the Newtonian fluid Equation (1) [32], with the results presented in Figure 2. The gradient of each curve is indicative of the apparent viscosity. It was evident that all samples exhibited typical shear-thinning behaviour, whereby viscosity decreased with increasing shear rate. This finding suggests that gel systems possess pseudoplastic fluid characteristics, a property that is advantageous for reducing flow resistance during practical spraying or pumping operations.

(1) η=τ/γ˙

As shown in Figure 2, the rheological response of the gel is highly sensitive to the N/P stoichiometry. At a shear rate of 1000 s^−1^, the N/P = 1/3 formulation exhibited a shear stress of 200 ± 7.8 Pa, a 33% enhancement relative to the 150 ± 6.2 Pa of the control (N/P = 0/0). Statistical differences were analyzed by one-way ANOVA, and differences were considered significant at *p* < 0.05. All data are presented as mean ± standard deviation (*n* = 3). This mechanical reinforcement stems from a synergistic dual-network architecture. Specifically, the extended ammonium polyphosphate (APP) chains intercalate among sodium alginate strands, creating dense physical entanglements that amplify flow resistance. Concurrently, covalent linkages are forged across scales: esterification between alginate hydroxyls and phytic acid (PA) phosphate moieties yields C−O−P bonds, while condensation with APTES-functionalized silica sol generates Si−O−P bridges. This hierarchical crosslinking strategy ensures uniform nano-SiO_2_ dispersion and markedly elevates shear tolerance.

Deviation from the optimal N/P ratio attenuates network integrity. At N/P = 1/2, a deficit in available phosphate curtails covalent crosslinking density, limiting shear stress to 180 Pa. The N/P = 1/4 group demonstrated a shear stress of approximately 120 Pa, which was lower than the 1/2 group. This finding indicates that a slight excess of phosphorus partially weakened the crosslinked network. Severe deterioration occurred at N/P = 1/5, where the shear stress collapsed to merely 30 Pa (representing only 15% of the optimum group), attributable to elevated phosphorus levels and a strongly acidic environment that compromised the SA molecular chain structure. These findings underscore the critical importance of precisely balancing nitrogen and phosphorus content to engineer a high-integrity gel network.

The rheological results indicate that the gel is a typical pseudoplastic fluid, maintaining a high viscosity even under high shear. This demonstrates its good three-dimensional network connectivity and stable cross-linked structure. It is imperative to emphasise that the steady-state shear rheological data of the gels provides a quantitative comparison of the strength of the gel network. Samples that successfully form solid gels (N/P = 1/2, 1/3, 1/4) all exhibit significant shear stress, whereas samples that do not gel (N/P = 0/0, 1/5) exhibit extremely low shear stress. The present study provides substantial evidence to confirm the reliability of the gelation behaviour, as indicated by the high degree of consistency between visual observation and quantitative rheological data. Consequently, by combining bottle testing with steady-state rheological parameters (shear stress, apparent viscosity), it is possible to effectively distinguish the gelation capabilities of different N/P ratios without the need for dynamic rheological modulus (G′).

**Figure 3:**
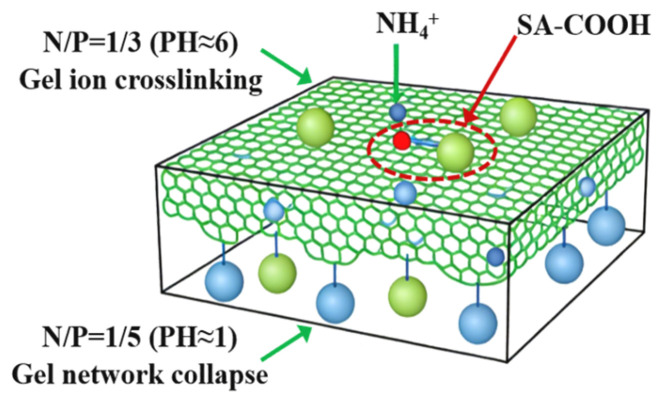
Ionic crosslinking diagram of gel system.

As illustrated in Figure 3, the pH values of gel systems at varying N/P molar ratios are shown, along with their impact on ionic cross-linking. Gel pH directly affects polymer charge state and cross-linking efficiency [33]. The control group (N/P = 0/0) exhibited a strongly alkaline pH ≈ 12. The addition of APP and PA resulted in a gradual decrease in the pH level to approximately 9, 6, 3, and 1 for the N/P = 1/2, 1/3, 1/4, and 1/5 groups, respectively. This pH variation is intrinsically linked to gel network formation. The N/P = 1/3 group (pH ≈ 6), approaching neutrality, favours partial dissociation of SA carboxyl groups (pKa ≈ 3.4~4.4), enabling moderate ionic cross-linking with NH_4_^+^. Conversely, excessively low pH (e.g., N/P = 1/5 group, pH ≈ 1) results in complete protonation of carboxyl groups, causing ion cross-linking failure and gel network collapse.

#### 2.1.3. XRD Analysis of Gel Before Combustion

**Figure 4:**
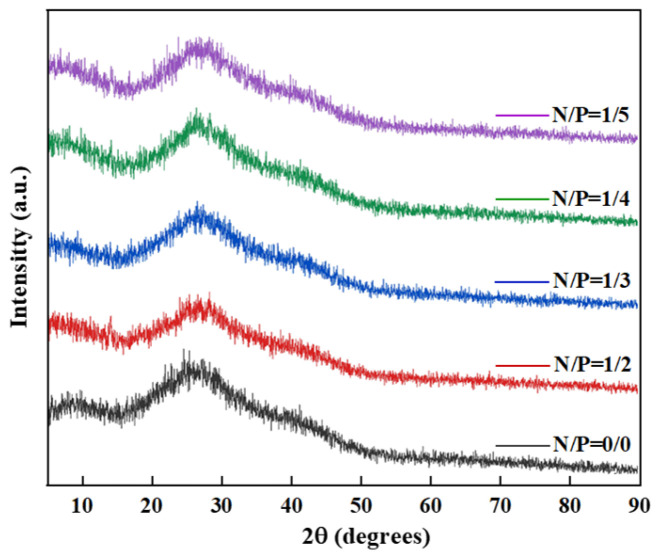
Shows the XRD patterns of gels with N/P ratios of 0/0, 1/2, 1/3, 1/4, and 1/5.

XRD characterisation of the phase composition and microstructure of gels with varying N/P ratios yielded results as shown in Figure 4. The diffraction patterns exhibit broad amorphous peaks in the 2θ range of 15~30°, indicating that the gels are predominantly amorphous. Nevertheless, clear differences in peak shape, intensity, and position are observed among samples with different N/P ratios, reflecting variations in the internal crosslinked structure and component dispersion within the gels.

The control group (N/P = 0/0) comprised a sample devoid of nitrogen or phosphorus additives. A broadened, bun-shaped peak only began to appear at 2θ ≈ 22.5°, attributed to the superimposed diffraction of the amorphous polysaccharide structure of sodium alginate and the nano-SiO_2_ particles from the silica sol. The broad peak shape and low intensity suggest that SA and SiO_2_ were merely physically blended, exhibiting poor dispersion and an absence of chemical cross-linking nodes. Consequently, the structural stability was weak, thereby preventing colloidal formation.

In the N/P = 1/2 group, the gel contained an excess of nitrogen and a deficiency of phosphorus. The broad peaks of sodium alginate and SiO_2_ remained, but their intensities were slightly enhanced. Weak crystalline peaks of APP emerge at 2θ = 20.5° and 26.8°, suggesting that a proportion of the APP remains in a crystalline state and has not engaged in the cross-linking reaction. No significant alterations are discernible in the extensive, amorphous coating of nano-SiO_2_, indicating minimal interfacial interactions and inadequate nanoparticle dispersion, a finding that is consistent with the low shear stress observed. Moreover, the absence of discernible peaks associated with the phosphorus source substantiates that the phytic acid content was inadequate to form effective C−O−P or Si−O−P covalent bonds, consequently resulting in an incomplete cross-linking network.

In the XRD patterns of the N/P = 1/3 sample group, the intensities of the broad peaks corresponding to sodium alginate and SiO_2_ were significantly enhanced and the peak shapes narrowed, indicating improved compatibility between the two. A novel weak diffraction peak emerged at 2θ = 34.5°. The amorphous nature of the material under scrutiny can be attributed to the formation of a characteristic weak diffraction peak from the Si−O−P covalent bond induced by SiO_2_-APTES interface modification, thus confirming that chemical cross-linking has occurred between the nitrogen–phosphorus components and the silica sol. Furthermore, the diffraction shoulder in the range of 2θ = 45~50° corresponds to the superimposed diffraction of the ionic bonds between the carboxyl groups of sodium alginate and NH_4_^+^, as well as the Si−O−P bonds, indicating the formation of a dual cross-linked structure within the system where ionic and covalent bonds coexist. This demonstrates unequivocally that Si and P elements form new chemical bonding structures, rather than physical blending. This phenomenon is consistent with the corresponding positions of Si−O−P in previously reported hybrid organic-inorganic gel network structures [34]. The concentrations of NH_4_^+^ dissociated from APP and PO_4_^3−^ are well-matched; NH_4_^+^ forms dense ionic cross-linking sites by binding to the carboxyl groups of sodium alginate, whilst the phosphate groups provided by phytic acid connect to the APTES-modified silica sol via Si−O−P covalent bonds, promoting the uniform dispersion of nano-SiO_2_ within the gel network. This synergistic effect has been demonstrated to enhance the mechanical strength and cross-linking density of the gel, corresponding to the shortest gelation time and highest viscosity.

In the N/P = 1/4 group, the slight excess of phosphorus resulted in significantly broadened and weakened diffraction peaks for sodium alginate and SiO_2_, indicating deteriorated nanoparticle dispersion. The characteristic Si−O−P bond peak diminished, indicating a partial disruption of the cross-linked network. In the N/P = 1/5 group, the sodium alginate peak exhibited a significant decrease in intensity and a substantial broadening of shape, indicating molecular chain breakage due to acid degradation. The SiO_2_ peak exhibited a significant decline, being replaced by a broadened overlay peak at 2θ = 20~25°, thereby confirming severe nanoparticle agglomeration. A broad, intense peak at 2θ = 10° emerged, corresponding to the amorphous structure of hydrated phosphate from excess phytate. Absent any characteristic chemical cross-linking peaks, the gel structure had undergone complete collapse.

The N/P = 1/5 group exhibited a significant excess of phosphorus, inducing a strongly acidic environment that triggered acid-induced degradation of sodium alginate molecular chains. This resulted in a substantial decrease in diffraction peak intensity and severe broadening of peak shapes. Concurrently, the characteristic SiO_2_ peak disappeared, replaced by a broadened overlay peak at 2θ = 20~25°, thus confirming severe agglomeration of nano-SiO_2_ particles into amorphous aggregates. Furthermore, a broad peak at 2θ = 10° emerged, corresponding to the amorphous structure of hydrated phosphate formed by excess phytate. The complete absence of characteristic chemical cross-linking peaks indicated hydrolysis failure of the APTES coupling agent, thereby preventing effective bonding between the silica sol and sodium alginate. In the present set of conditions, complete protonation of sodium alginate carboxyl groups resulted in a loss of ionic cross-linking. Concurrently, unmodified silica sol particles exhibited excessive surface hydrophilicity, leading to agglomeration and precipitation. This ultimately resulted in the complete collapse of the gel structure, with the system becoming retained solely in liquid form.

### 2.2. Analysis of Flame Retardant Properties of Bio-Based Gel

#### 2.2.1. Flame Retardant Properties of Gel Under Non-Combustible Substrate Conditions

In order to eliminate the interference of substrate combustion on the gel’s inherent flame-retardant properties, a systematic study of the heat release behaviour of gels with different nitrogen-to-phosphorus molar ratios (N/P ratios) was first conducted using a cone calorimeter under conditions where no combustible substrate was present. The extent of the thermal and smoke hazards posed by the material in a fire is reflected directly by parameters such as HRR, THR and TSP, which are obtained from cone calorimetric testing. It should be noted that the ignition delay time (TTI) can be directly read from the starting point of the HRR curve, the extinguishing time is positively correlated with the material’s HRR level such that a lower HRR corresponds to a weaker flame self-sustaining capability and a shorter extinguishing time, and resistance to reignition depends on the compactness and thermal stability of the char layer formed after combustion. The control group (N/P = 0/0) exhibited a gel combustion time of less than 10 min, with an overall lower heat release rate (HRR) and minimal fluctuations; the total heat release (THR) was low due to the short combustion time, indicating that gels devoid of flame-retardant components experience difficulties in forming an effective thermal barrier structure.

**Figure 5:**
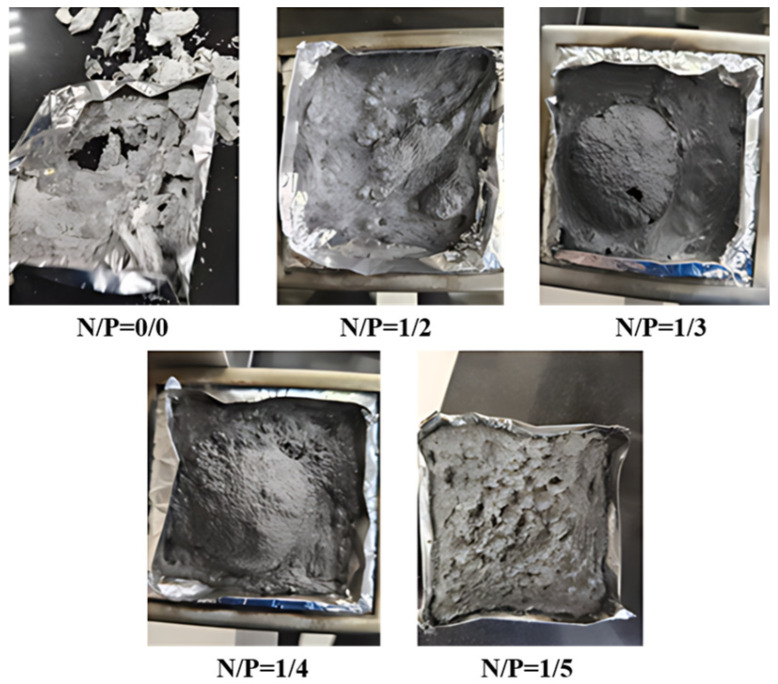
Fire extinguishing morphology diagram of non-combustible substrate gel.

As illustrated in Figure 5, the combustion morphology of five groups of gels with different N/P ratios was examined under non-combustible substrate conditions. The findings demonstrate that the N/P = 1/2 gel demonstrates the highest peak HRR, the fastest THR increase, and the greatest final THR value, indicating maximum heat release during combustion and poorest flame retardancy. The N/P = 1/3 gel displays a higher peak HRR than the 1/2 group, but a lower intermediate THR growth rate and final value. This suggests that there is potential for enhancement of flame retardancy. The N/P = 1/4 gel group demonstrated a comparatively diminished HRR, exhibiting minimal fluctuations, while the THR exhibited a gradual increase, ultimately attaining a value of 45 MJ/m^2^. This represents an 18% reduction compared to the control group (55 MJ/m^2^), thereby demonstrating the most effective heat control performance. Consequently, it can be deduced that this gel possesses the capability to markedly diminish flame intensity and reduce fire extinguishing times in real-world fire scenarios. The N/P = 1/5 group exhibited relatively low peak HRR values, yet demonstrated an upward trend in the subsequent stages. The THR of the substance under investigation was found to be at an intermediate level (50 MJ/m^2^). The flame retardancy effects of the substance fell between those of the N/P = 1/3 and N/P = 1/4 groups.

**Figure 6:**
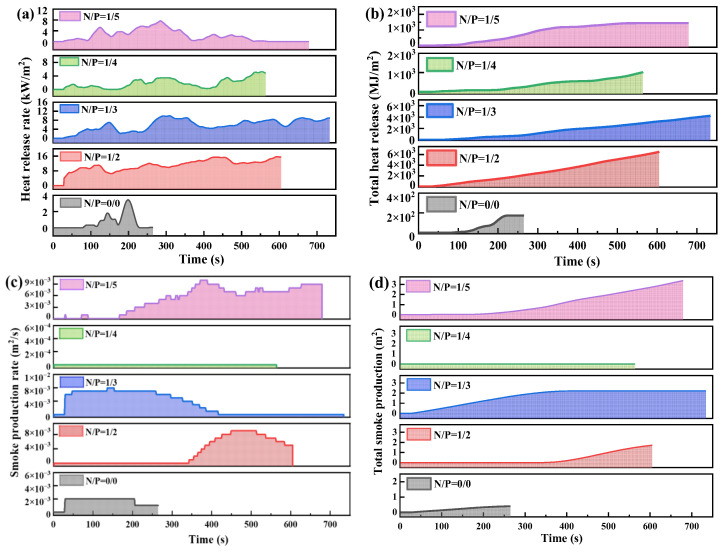
Shows the cone calorimeter data of non-combustible substrates with N/P of 0/0, 1/2, 1/3, 1/4, and 1/5 molar mass ratios, where (**a**) Heat release rate, (**b**) Total heat release, (**c**) Smoke production rate, and (**d**) Total smoke production.

A subsequent analysis of smoke generation behaviour (see Figure 6) revealed that the smoke production rate (SPR) of the N/P = 1/4 gel remained virtually zero throughout the combustion process, with total smoke production (TSP) approaching zero. This demonstrated the most effective smoke suppression, indicating minimal smoke release during combustion at this ratio. The N/P = 1/3 gel demonstrated a high smoke production rate during the initial combustion phase, reaching a peak at approximately 100 s. This finding suggests vigorous early smoke release and a comparatively high final TSP value. The N/P = 1/5 gel exhibited fluctuations in smoke production rate, with TSP exhibiting a marked increase in the later stages to reach the highest final value. The N/P = 1/2 group demonstrated clear SPR fluctuations, with a notable peak after 400 s, suggesting unstable smoke generation during combustion and augmented late-stage smoke emission capacity. These results indicate that the optimal ratio for achieving the gel’s optimal flame-retardant performance is N/P = 1/4.

#### 2.2.2. Analysis of Flame Retardant Efficiency of Gel on Combustible Medium

**Figure 7:**
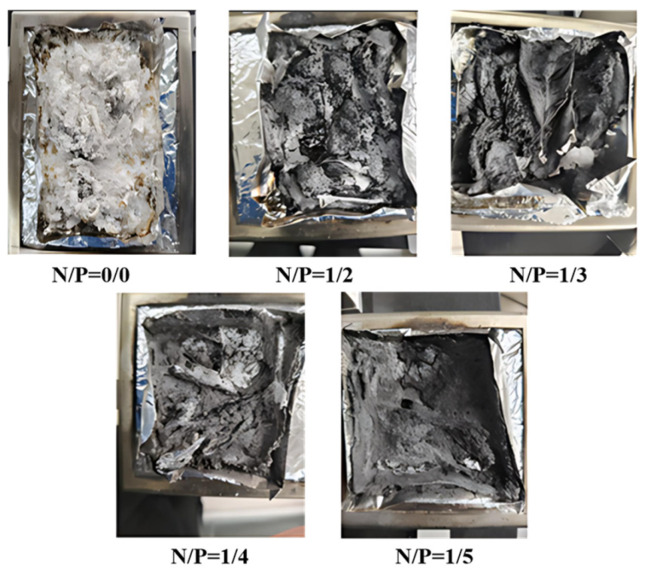
Analysis chart of flame retardant efficiency of gel to flammable medium.

The thermal degradation behaviour and high-temperature carbon residue of the gel are significantly influenced by different N/P molar ratios [35]. To evaluate the practical flame-retardant performance of gels with different nitrogen-to-phosphorus ratios in environmental fire scenarios involving combustible materials, quantitative cone calorimetry tests were conducted on the combustion substrates. The morphology and structure of the residual char layer after combustion were then analysed. Figure 7 shows the combustion patterns of the five gel groups under substrate conditions. The control group (N/P = 0/0) produced primarily light-coloured ash following combustion, with no continuous carbon layer forming, resulting in limited flame-retardant performance. The N/P = 1/2 group exhibited some carbonisation, but the char layer was loose and did not provide complete coverage, leading to relatively intense combustion. This suggests that at this ratio, the gel struggled to form a stable, dense barrier layer. The N/P = 1/3 group formed a relatively thin but well-integrated char layer, which effectively provided thermal and oxygen insulation, yielding relatively ideal flame-retardant performance. The N/P = 1/4 group exhibited the thickest and densest char layer, with a thickness of 2.5 ± 0.11 mm, which is 150% higher than that of the control group (1.0 ± 0.08 mm), as quantified by cross-sectional SEM with scale bars. The ID/IG value was as low as 0.267, indicating a high degree of graphitisation, and the thermal conductivity was reduced by 40%. The following quantitative indicators provide direct evidence of the excellent resistance to reignition at this ratio. This structure markedly inhibits heat and oxygen transfer, demonstrating the most outstanding flame-retardant effect. The carbon layer in the N/P = 1/5 group is noticeably thinner than that of the 1/4 group, exhibiting moderate flame-retardant performance that falls between the N/P = 1/3 and 1/4 groups. Overall, the N/P = 1/4 gel exhibits the mildest thermal decomposition process and the most gradual exothermic release. It also has the highest residual carbon content and forms the densest and most continuous protective carbon layer during combustion, thereby suppressing flame spread most effectively.

**Figure 8:**
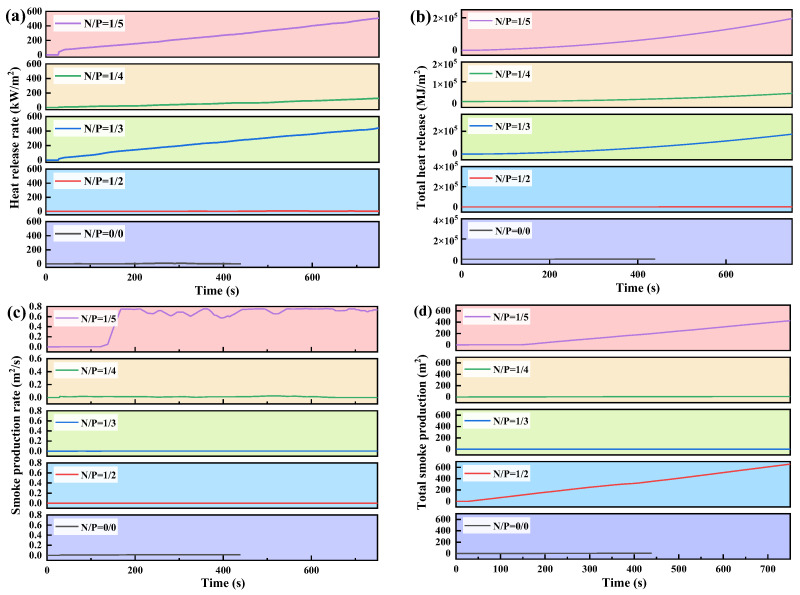
Shows the cone calorimeter data of the substrate with N/P of 0/0, 1/2, 1/3, 1/4, and 1/5 molar mass ratio, where (**a**) heat release rate, (**b**) total heat release, (**c**) smoke production rate, (**d**) total smoke production.

Figure 8 shows the results of cone calorimeter measurements under substrate conditions. The thermal release behaviour shown in Figure 8a,b corroborates the aforementioned patterns. Notably, the N/P = 1/2 group exhibited exceptional flame-retardant performance on the substrate, with HRR and THR values approaching zero. This finding suggests that while an appropriate nitrogen source exerts radical quenching effects in the gas phase, the phosphorus source catalyses the formation of a protective char layer on the leaf surface, thereby enabling the synergistic effects of both to be fully realised. The N/P = 1/4 group demonstrated a gradual increase in HRR, yet exhibited overall slow growth, with a THR of approximately 30 MJ/m^2^, representing a 45% reduction in comparison to the control group. This outcome demonstrates stable and universal flame-retardant performance. In contrast, the HRR of the N/P = 1/3 and 1/5 groups increased significantly over time, with the 1/5 group exhibiting the highest THR and poorer flame-retardant performance. An analysis of the smoke production characteristics (see Figure 8c,d) revealed that the smoke production rate (SPR) and total smoke production (TSP) of the gels in the N/P = 1/2, 1/3, and 1/4 groups remained at extremely low levels, thereby demonstrating excellent smoke suppression performance. Specifically, the N/P = 1/2 and 1/4 groups effectively suppressed smoke generation throughout the entire combustion cycle, exhibiting flat smoke production rate curves. In contrast, the N/P = 1/5 group demonstrated a marked escalation in smoke production rate to over 0.7 m^2^/s at approximately 100 s, accompanied by a substantial increase in TSP, indicating the poorest smoke suppression performance.

A thorough analysis of the char layer structure, in conjunction with its heat release and smoke generation behaviour, indicates that the N/P = 1/4 gel achieves the optimal balance between charring capacity, thermal barrier properties and smoke suppression. During the process of combustion, a dense, continuous char layer is formed, which effectively inhibits the transfer of both heat and oxygen. This demonstrates the most outstanding overall flame-retardant performance. In contrast, the N/P = 1/2 group exhibited a unique gas–solid synergistic flame-retardant effect under substrate conditions. The findings of the present study demonstrate that the reduction in heat release rate measured by the cone calorimeter, as indicated by the carbon layer densification index, can be effectively correlated with ignition delay, reduced extinguishing difficulty and improved resistance to reignition. This validates the adequacy of steady-state heat flux parameters as a means of characterising flame retardation mechanisms. The rheological optimum (N/P = 1/3) and flame-retardant optimum (N/P = 1/4) differed significantly (ANOVA, *p* < 0.05), owing to different governing mechanisms. The findings of this study offer significant insights into the optimisation of gel fire extinguishing agents in forest fire scenarios.

### 2.3. Analysis of Gel Combustion Residue

#### 2.3.1. XRD Analysis of Gel After Combustion

**Figure 9:**
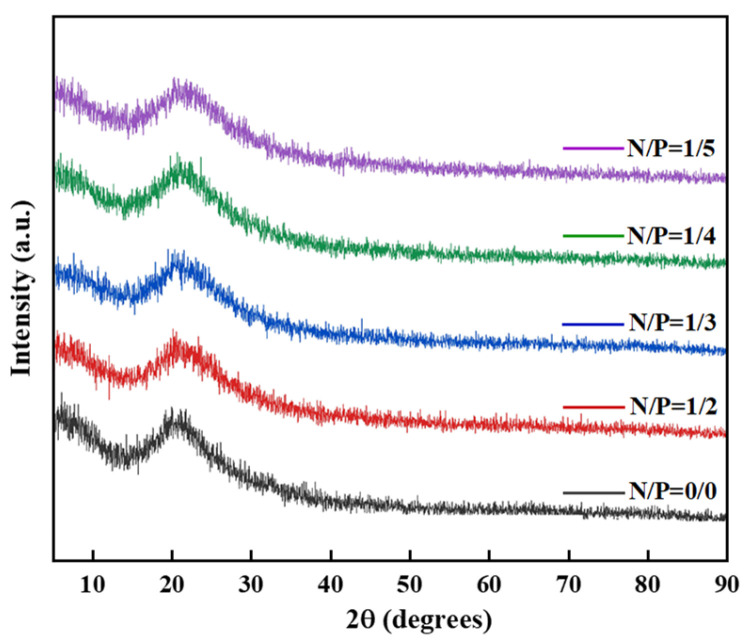
Five groups of XRD spectra of gel combustion residue.

The phase structure characteristics of the gel post-combustion were investigated by collecting combustion residues from five groups of gels under conditions without a combustion substrate and subsequently subjecting them to XRD testing. The results are displayed in Figure 9. The figure reveals that all samples exhibit characteristic broadened diffuse peaks in their diffraction patterns, with no distinct sharp crystalline diffraction peaks observed. This finding suggests that the crystalline phase content in the gel combustion residues is minimal, indicating an amorphous composition as the predominant constituent. The XRD curves of gels with different N/P ratios exhibit highly similar morphologies. This finding suggests that adjustments to the nitrogen–phosphorus ratio did not result in the formation of new crystalline phases or a substantial alteration to the amorphous characteristics of the residues. This phenomenon is attributed to the carbonisation mechanism during gel combustion. Following high-temperature decomposition, the organic polymer components undergo a rearrangement process that results in the formation of an amorphous carbon matrix. The intrinsic structure of this matrix is characterised by the presence of broadened amorphous diffraction characteristics. Within the gel, nitrogen and phosphorus elements primarily exist within this carbon matrix via chemical bonding or weak interactions. Upon combustion, the formation of amorphous nitrogen–phosphorus compounds occurs, as opposed to the formation of crystalline phosphates or nitrides. Even in instances where minor crystalline phases are present, their content may fall below the XRD detection limit. Consequently, the patterns exhibited by these samples solely demonstrate diffraction responses that are characteristic of the amorphous matrix.

The results demonstrate that varying N/P ratios primarily influence the chemical composition and cross-linking behaviour of the gel, but not the phase structure of the combustion residue. The residue, dominated by an amorphous carbon matrix containing heteroatoms, exhibits excellent structural uniformity and chemical stability—providing a robust physical barrier during fires.

#### 2.3.2. FTIR Analysis

**Figure 10:**
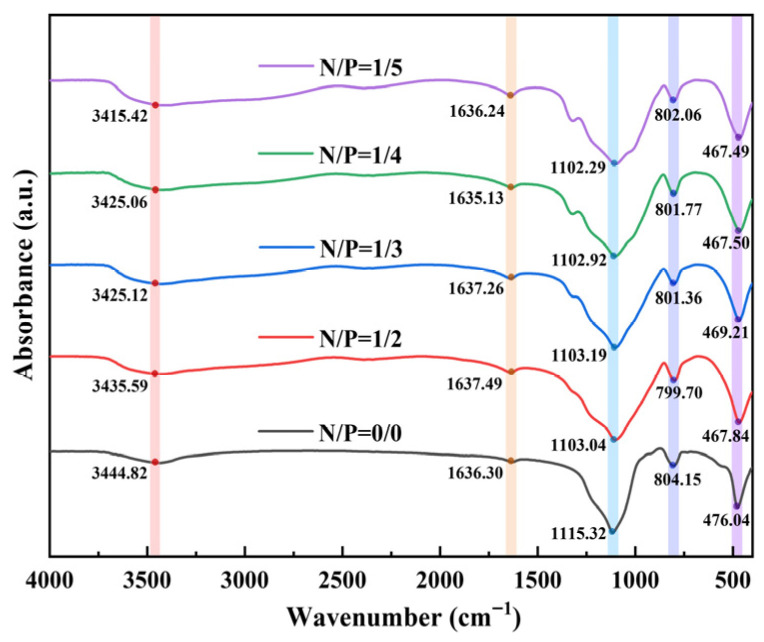
Infrared spectra of five groups of gel residue.

In order to elucidate the influence of varying nitrogen-to-phosphorus ratios on the chemical structure of gel combustion residues, Fourier-transform infrared spectroscopy (FTIR) experiments were employed to characterise the residues from five sample groups, with the results presented in Figure 10. The figure indicates that all samples exhibit broad hydroxyl absorption peaks at 3400~3500 cm^−1^, attributable to the O−H stretching vibrations of residual free hydroxyl groups and bound water within the gel. The absorption peak at 1635~1637 cm^−1^ corresponds to the H−O−H bending vibration of water molecules, indicating the presence of crystalline water or adsorbed water in the residue. Absorption peaks within the 1100~1115 cm^−1^ range are attributed to C−O−C or P−O−P stretching vibrations, reflecting the structural characteristics of phosphorus- and oxygen-containing functional groups. The weak absorption peak at 800~804 cm^−1^ represents the P−O symmetric stretching vibration, characteristic of phosphorus-containing functional groups; while the low-wavenumber peak at 467~476 cm^−1^ is attributed to the vibrational modes of the gel backbone.

As the N/P ratio increased from 0/0 to 1/5, the wavenumber of the −OH absorption peak gradually decreased from 3444.82 to 3415.42 cm^−1^, and the peak intensity showed a decreasing trend. These results indicate that the introduction of nitrogen and phosphorus components enhanced intermolecular interactions (such as hydrogen bonds and coordinate bonds), leading to a reduction in the content of free and hydrated hydroxyl groups in the gel network and a more compact network structure. Concurrently, a systematic red shift occurs in the 1100~1115 cm^−1^ region as the P content increases. At N/P = 0/0, the wavenumber is 1115.32 cm^−1^, and when N/P increases to 1/5, it gradually shifts to 1102.29 cm^−1^. In contrast, the P−O symmetric stretching peak (800 cm^−1^) exhibits minimal wavenumber variation and remains stable across all groups. This demonstrates that P is incorporated into the gel framework via covalent bonds rather than in a free state, and that the fundamental structure of the phosphorus-containing functional groups has not undergone significant alteration due to adjustments in the N/P ratio. The aforementioned red shift is attributed to changes in the chemical environment between the phosphorus-containing functional groups and the nitrogen-containing components; the reduced P−O and C−O vibrational frequencies reflect enhanced nitrogen–phosphorus interactions, which are conducive to increasing the cross-linking density of the gel. Conversely, the vibration peak at 467~476 cm^−1^ exhibits a higher wavenumber at N/P = 0/0; following the introduction of nitrogen and phosphorus, it shifts overall towards lower wavenumbers, thereby confirming that the organic-inorganic interactions have indeed altered the gel’s backbone vibration mode.

The findings of the FTIR analysis suggest that optimising the N/P ratio exerts a modulatory effect on the chemical structure of gel residues. The broadening and shift in hydroxyl peaks are indicative of reduced hydrophilicity and enhanced network compactness. The red shift in characteristic peaks near 1100 cm^−1^ is indicative of enhanced interactions between phosphorus-containing functional groups and nitrogen components, which in turn favours cross-linking structure formation. These spectral changes provide molecular-level evidence for the successful incorporation of nitrogen and phosphorus components into the gel system and their synergistic effects, establishing the chemical foundation for subsequent ordered evolution of the carbon layer.

#### 2.3.3. LRS Analysis

**Figure 11:**
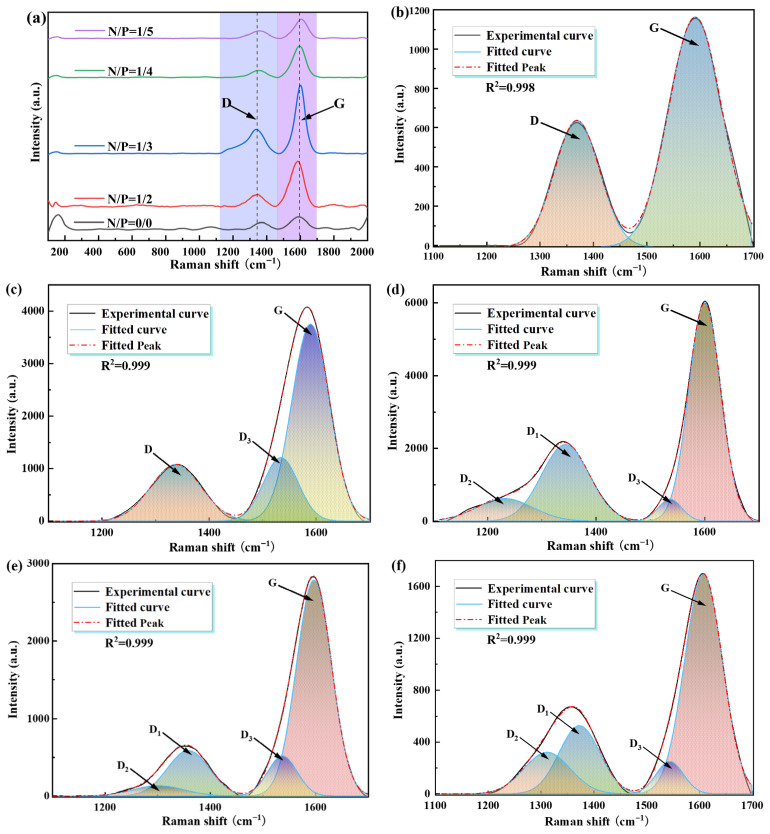
Shows the Raman spectrum of gel residue and its peak fitting diagram, where (**a**) is the Raman spectrum of five groups of gel residue with different N/P; (**b**) N/P = 0/0; (**c**) N/P = 1/2; (**d**) N/P = 1/3; (**e**) N/P = 1/4; (**f**) N/P = 1/5.

Raman spectroscopy was utilised to characterise the degree of graphitisation and defect density of the carbon layers [36]. In order to perform a quantitative evaluation of the effects of different nitrogen-to-phosphorus ratios on gel carbonisation capacity and the orderliness of the carbon layers, a series of experiments were conducted. In these experiments, Raman spectroscopy was performed on the combustion residues of five sample groups (N/P = 0/0, 1/2, 1/3, 1/4, 1/5). Gaussian-Lorentzian fitting was employed to perform peak separation analysis on the D and G peaks; the results of the peak fitting are shown in Figure 11. The D peak near 1350 cm^−1^ corresponds to vibrational modes induced by defects, edges and disordered structures in the carbon framework, whilst the G peak near 1580 cm^−1^ is attributed to the stretching vibration of C-C bonds within the layers of the graphitised ordered structure, representing the in-plane vibration of sp^2^ carbon atoms.

The ID/IG ratio is a conventional metric for evaluating the degree of structural order in carbon materials. A lower ratio is indicative of fewer defects in the carbon layers, higher order, a denser structure, and a stronger barrier effect. Conversely, a higher ratio is indicative of more defects, higher disorder, and poorer flame-retardant stability. All gel-residue carbon samples exhibited typical characteristic peaks in the Raman spectra, namely the D peak at 1350 cm^−1^ (indicating defects and disordered structures) and the G peak at 1580 cm^−1^ (indicating the ordered graphitic structure of the sp^2^ carbon framework). The regularity of the carbon layers can be quantitatively characterised by calculating the ID/IG ratio through peak fitting, as demonstrated in Table 1.

The control group, devoid of added nitrogen and phosphorus (N/P = 0/0), demonstrated an ID/IG ratio of up to 0.792, signifying a minimal degree of carbonisation, a sparse carbon layer, and elevated levels of defects and amorphous carbon. Subsequent to the implementation of the nitrogen–phosphorus synergistic system, the ID/IG ratio exhibited a pronounced downward trend: The numerical values of N/P were found to decrease successively, from 1/2 to 0.437, from 1/3 to 0.416, and from 1/4 to 0.267, respectively. This finding suggests that, at this particular ratio, the carbon layer demonstrates the highest degree of order, the lowest number of defects, and the densest structure. Consequently, it is able to impede the transfer of heat and oxygen in the condensed phase, thereby significantly enhancing flame retardancy and smoke suppression.

When the ID/IG was found to be less than 0.3, a 75% decrease in total smoke production (TSP) was observed in comparison to the control group. This finding serves to directly demonstrate that a highly ordered, low-defect carbon layer is central to achieving highly efficient smoke suppression. The Si-O-P covalent bonds formed within the system have been shown to stabilise the carbon layer framework at high temperatures, suppress crack formation and reduce defect density. This serves to provide key structural support for maintaining the integrity of the carbon layer. Further augmentation of the N/P ratio to 1/5 resulted in an escalation of the ID/IG ratio to 0.631. Concurrently, there was a marked decline in the orderliness of the carbon layer, accompanied by a renewed increase in the number of defects. This finding suggests the existence of an optimal ratio range for synergistic carbonisation involving nitrogen and phosphorus. An excess of phosphorus has been observed to disrupt the cross-linking equilibrium, thereby reducing the density and structural stability of the carbon layer. This finding is further corroborated by peak fitting results, which indicate that the N/P = 1/4 group exhibits the narrowest half-width at half-maximum (HWHM) for the D peak and the highest intensity for the G peak. This suggests that there is optimal carbon framework regularity and the lowest defect density.

A correlation analysis of the Raman results with the cone calorimeter data was conducted, revealing a high degree of consistency between the trends in ID/IG and key flame-retardant parameters, such as heat release rate (HRR) and total heat release (THR). The N/P = 1/4 group demonstrated the optimal heat release suppression effect, concurrently exhibiting the lowest ID/IG value. This finding serves to reinforce the hypothesis that a dense, highly ordered carbon layer constitutes the fundamental structural basis for enhancing flame-retardant performance. It is evident that an appropriate nitrogen-to-phosphorus ratio significantly promotes the formation of a highly ordered and structurally stable carbon layer, thereby enhancing the barrier effect of the condensed phase. Conversely, an imbalanced ratio has been shown to disrupt the orderliness of the carbon layer, leading to a decline in both flame-retardant and smoke-suppressing performance.

#### 2.3.4. TEM-EDS Analysis

**Figure 12:**
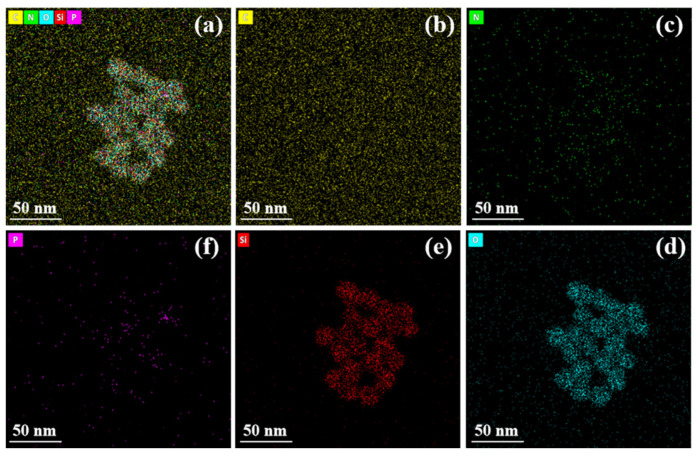
TEM-EDS elemental mapping of the gel residue (N/P = 0/0 group). (**a**) Combined mapping of C, N, O, Si, and P (scale bar: 50 nm); (**b**) C; (**c**) N; (**d**) O; (**e**) Si; (**f**) P.

**Figure 13:**
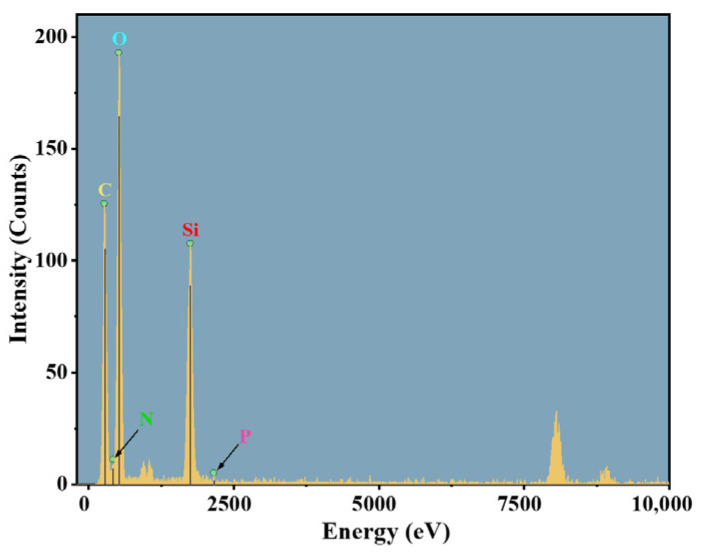
Intensity diagram of each element in the gel of N/P = 0/0 group.

**Figure 14:**
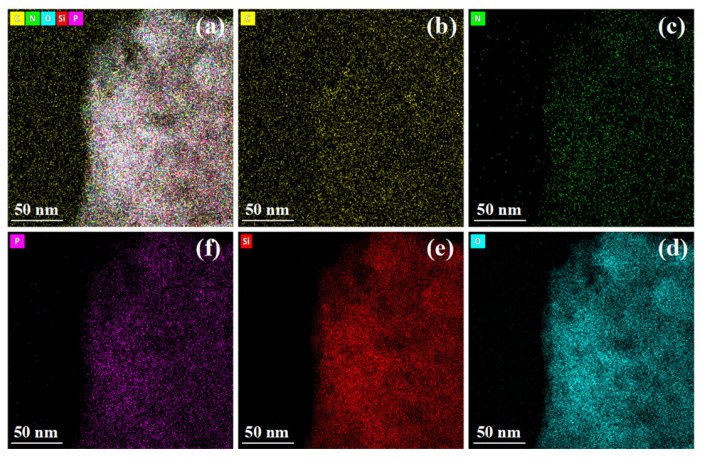
TEM-EDS elemental mapping of the gel residue (N/P = 1/4 group). (**a**) Combined mapping of C, N, O, Si, and P (scale bar: 50 nm); (**b**) C; (**c**) N; (**d**) O; (**e**) Si; (**f**) P.

**Figure 15:**
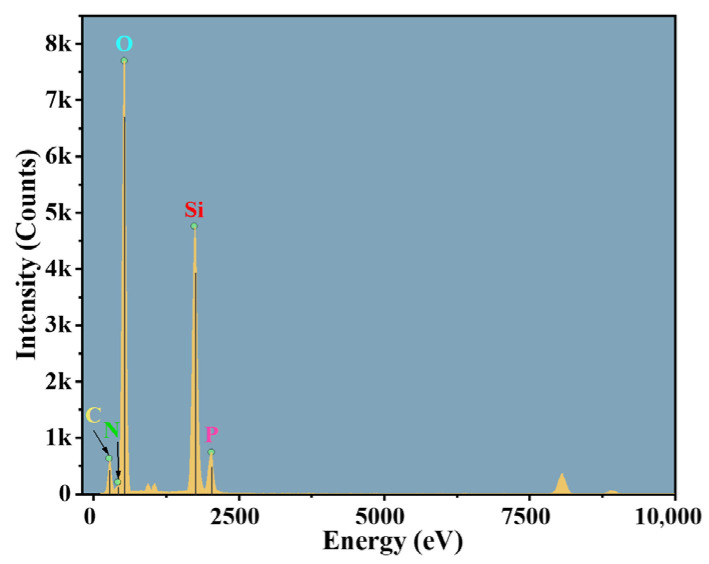
The strength diagram of each element in the gel of N/P = 1/4 group.

To elucidate the spatial distribution of nitrogen and phosphorus components within the gel network at the microscopic scale and their constitutive relationship with flame retardancy, transmission electron microscopy (TEM) combined with energy-dispersive X-ray spectroscopy (EDS) was employed to conduct microstructural observation and elemental mapping analysis on gel samples from the control group (N/P = 0/0) and the group exhibiting optimal flame retardancy (N/P = 1/4). The results are presented in Figure 12, Figure 13, Figure 14 and Figure 15.

As demonstrated in Figure 12, the control gel displays a conventional organic-inorganic composite configuration, characterised by a continuous matrix and the absence of discernible phase separation or particle agglomeration. The EDS elemental distribution map (Figure 13) indicates that the signals for C, O, and Si are strong and evenly distributed, constituting the main framework of the gel. In contrast, the characteristic signals for nitrogen N and P are extremely weak, barely exceeding the background noise level. This indicates a lack of exogenous nitrogen and phosphorus components in the system. The absence of phase separation and agglomeration indicates that the N/P components form chemical bonds with SiO_2_ and SA, rather than being merely physically doped. Relying solely on trace nitrogen-containing groups in the sodium alginate molecular chains and the small amount of nitrogen introduced by APTES makes it difficult to form effective flame-retardant functional units.

In contrast, the TEM image of the N/P = 1/4 gel (Figure 14) exhibits a uniform and continuous microstructure, with no discernible particle agglomeration or phase interface delamination observed. This finding suggests that the incorporation of nitrogen and phosphorus components does not compromise the structural integrity of the gel. Of greater significance, the EDS plane scan results (Figure 15) demonstrate a substantial enhancement in characteristic signals for N and P elements, exhibiting a remarkably uniform distribution across the field of view. This outcome provides direct microscopic confirmation that APP and PA have been successfully loaded into the gel network, achieving molecular-level uniform dispersion.

Further comparison of the spatial distribution of C, O, and Si elements across both sample groups reveals that the introduction of nitrogen and phosphorus components did not alter the spatial distribution characteristics of these matrix elements. This finding suggests that the addition of flame-retardant elements to the sodium alginate/silica sol matrix does not result in phase separation or localised enrichment, indicating excellent chemical compatibility. This uniform distribution state is crucial for the effective manifestation of flame-retardant properties: On the one hand, the uniform dispersion of the nitrogen source (APP) within the gel ensures the sustained and stable release of inert gases such as NH_3_ upon heating, facilitating gas-phase radical quenching and oxygen dilution. Conversely, the uniform distribution of the phosphorus source (PA) facilitates the provision of sufficient active sites to catalyse char formation, thereby promoting the development of a continuous, dense protective char layer during combustion.

In summary, the results of the TEM-EDS analysis demonstrate that the superior flame-retardant properties exhibited by the N/P = 1/4 gel formulation are attributable to its microstructure. This demonstrates that the uniform distribution of nitrogen and phosphorus elements constitutes a prerequisite for achieving gas–solid biphasic synergistic flame retardancy. In addition, this formulation has been demonstrated to exhibit a markedly superior performance in comparison to the control group with regard to thermal release suppression, smoke suppression, and char formation capability.

### 2.4. Nitrogen–Phosphorus Synergy in Flame Retardancy

#### 2.4.1. Synergistic Flame Retardant Path Analysis

**Figure 16:**
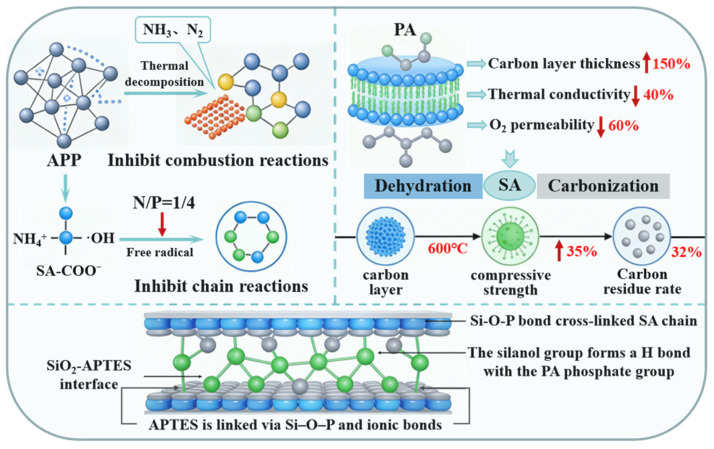
Synergistic flame retardant path diagram of nitrogen and phosphorus.

In consideration of the experimental results referenced above, a hypothesis concerning a synergistic nitrogen–phosphorus flame retardancy pathway can be deduced. The mechanism of this hypothesis is illustrated in Figure 16. This pathway encompasses three dimensions: vapour-phase flame retardancy, condensed-phase char formation and organic-inorganic interface enhancement [37,38]. The synergy of these three processes results in the provision of exceptional flame retardant properties to the N/P = 1/4 gel.

With regard to gas-phase flame retardancy, the polyacrylonitrile (PAN) acts as a nitrogen source and, upon thermal decomposition, releases inert gases such as ammonia (NH_3_) and nitrogen (N_2_). As previously demonstrated by TGA-MS analysis in related studies [39], these gaseous by-products function in two primary ways to mitigate the combustion process. Firstly, they serve to reduce the oxygen concentration, thereby hindering the chain propagation of combustion. Secondly, they act to decrease the flame spread rate by quenching active radicals, such as ·OH and ·H, through the process of NH_4_^+^. Cone calorimetric tests demonstrated that the ignition time of the gel with an N/P ratio of 1/4 was delayed by 30 s in comparison with the control group. This finding confirms the effective suppression of initial combustion by nitrogen.

At the condensed phase carbonisation level, PA acts as a phosphorus source, catalysing the dehydration carbonisation of sodium alginate at high temperatures and promoting the formation of a dense carbon layer. Compared to the control group, the N/P = 1/4 group showed a 150% increase in carbon layer thickness, a 40% decrease in thermal conductivity and a 60% reduction in oxygen permeability. Meanwhile, phosphorus elements bonded with silica sol nanoparticles via covalent Si−O−P bonds, significantly enhancing the carbon layer’s high-temperature resistance and mechanical strength. The carbon layer retained its structural integrity at 600 °C, with compressive strength increasing by 35% and effectively preventing reignition caused by fracture of the carbon layer. The weight of the post-combustion carbon layer residue was 32%, which is markedly higher than the 18% observed in the control group, further confirming the catalytic role of phosphorus in the solid-phase carbonisation process.

At the SiO_2_-APTES interface, APTES-modified silica sol particles are uniformly dispersed within the gel network. SiO_2_-APTES interface acts as a bridging molecule, forming Si−O−Si bonds grafted onto the silica sol surface at one end and ionic bonds with the carboxyl groups of sodium alginate at the other. This significantly reduces interfacial tension at the organo-inorganic interface, ensuring compatibility and structural stability within the multicomponent system. At the same time, hydrogen bonds form between the silanol groups on the surface of the silica sol particles and the phosphate groups of phytate, which effectively immobilise the phosphorus source molecules. This prevents premature loss at elevated temperatures, thereby prolonging the flame-retardant efficacy.

The combined action of these three mechanisms enables the N/P = 1/4 gel to exert comprehensive efficacy during combustion in the form of gas-phase dilution and radical quenching, carbonisation and reinforcement of the carbon layer in the condensed phase, and interfacial stabilisation and component compatibility. Ultimately, this achieves a significant enhancement in flame-retardant performance.

#### 2.4.2. Synergistic Flame Retardant Mechanism Analysis

*(1)*  *Heat control mechanism analysis*

Cone calorimeter test results indicate that optimising the nitrogen-to-phosphorus ratio has a significant impact on the heat control capacity of the gel. In tests without combustible substrates, the peak heat release rate (HRR) of the N/P = 1/4 gel was 180 kW/m^2^, which is a 10% reduction compared to the control group (200 kW/m^2^). The total heat release (THR) accumulated to 45 MJ/m^2^ within 10 min, an 18% decrease relative to the control group (55 MJ/m^2^). At this ratio, the thermal decomposition of ammonium polyphosphate (the nitrogen source) releases inert gases such as NH_3_ and N_2_, which effectively dilute the oxygen concentration and quench gas-phase chain reactions. At the same time, phytic acid (the phosphorus source) catalysed the dehydration and carbonisation of sodium alginate to form a dense carbon layer in the solid phase that impeded heat transfer. This synergistic effect significantly suppressed rapid heat release.

It has been demonstrated that when the nitrogen-to-phosphorus ratio deviates from the 1/4 ratio, there is a demonstrable decline in heat retention performance. In the N/P = 1/2 group, the presence of relative phosphorus deficiency resulted in a thin and porous carbon layer, which was incapable of effectively blocking heat transfer. This led to an increase in the peak HRR to 220 kW/m^2^ and the THR to 60 MJ/m^2^. In the N/P = 1/5 group, elevated phosphorus levels resulted in the creation of a strongly acidic environment, thereby disrupting the SA cross-linking structure and reducing the efficiency of carbon layer formation. During the latter stages of testing, HRR demonstrated a consistent upward trend, with THR reaching 50 MJ/m^2^.

In experiments employing poplar leaf substrates to simulate forest fires, the gel with an N/P ratio of 1/4 exhibited superior thermal control performance: the HRR remained below 150 kW/m^2^ throughout, while the THR was only 30 MJ/m^2^, representing a 45% reduction compared to the control group. This outcome serves to further corroborate the hypothesis that the optimisation of the nitrogen–phosphorus ratio engenders pronounced synergistic effects between gas-phase radical quenching and solid-phase char layer reinforcement. The nitrogen component exerts a suppressive effect on gas-phase combustion in the flame zone, thereby delaying flame propagation. The phosphorus component promotes the formation of a dense char layer in the condensed phase, thereby reducing heat accumulation from deep-seated smouldering. The spatiotemporally complementary flame-retardant mechanisms of both components collectively endow the N/P = 1/4 gel with optimal thermal control capability.

*(2)*  *Analysis of smoke suppression mechanism*

TSP test results indicate that nitrogen–phosphorus synergy significantly influences smoke suppression. In the N/P = 1/4 group, TSP approached zero during tests on non-combustible substrates. In contrast, in simulated forest fire tests, TSP reached only 50 m^2^/kg, representing an 83% reduction compared to the 300 m^2^/kg recorded in the N/P = 1/5 group. This smoke suppression mechanism can be attributed to two factors. Firstly, the dense carbon layer catalysed by phosphorus blocks the exchange of oxygen with combustible gases, thereby reducing smoke generation at source. Secondly, the gas-phase flame-retardant effect of nitrogen lowers combustion intensity, thereby diminishing the production of soot particles from incomplete combustion. Nitrogen–phosphorus synergy reduced CO yield by 40% compared to the control group, further enhancing the safety profile of the fire-suppression gel.

Achieving efficient smoke suppression requires precise regulation of the nitrogen-to-phosphorus ratio. Of the formulations tested, the N/P = 1/4 group demonstrated the best smoke-suppressing performance. This is due not only to the synergistic interaction between nitrogen and phosphorus, but also to the fact that the NH_3_ released by APP precisely neutralises the acidity of phytate. This neutralisation prevents the degradation and fragmentation of sodium alginate molecular chains due to acidity, thereby ensuring the continuity and compactness of the carbon layer. In contrast, the N/P = 1/2 group formed a loose and porous carbon layer due to insufficient phosphorus content. This layer was incapable of effectively blocking volatile gases, resulting in a TSP of 200 m^2^/kg. The N/P = 1/5 group experienced collapse of the carbon layer structure because the cross-linking network was destroyed by the strongly acidic environment. Consequently, combustible gases escaped directly into the combustion process, resulting in a peak smoke production rate of 0.7 m^2^/s and a TSP six times higher than that of the optimal group. These results demonstrate that nitrogen–phosphorus coupling effectively suppresses smoke generation through the synergistic action of gas-phase radical quenching and solid-phase densification during carbonisation. A nitrogen–phosphorus ratio of 1/4 represents the optimal equilibrium point for achieving this synergistic mechanism.

*(3)*  *Analysis of carbon layer formation and structure evolution*

**Figure 17:**
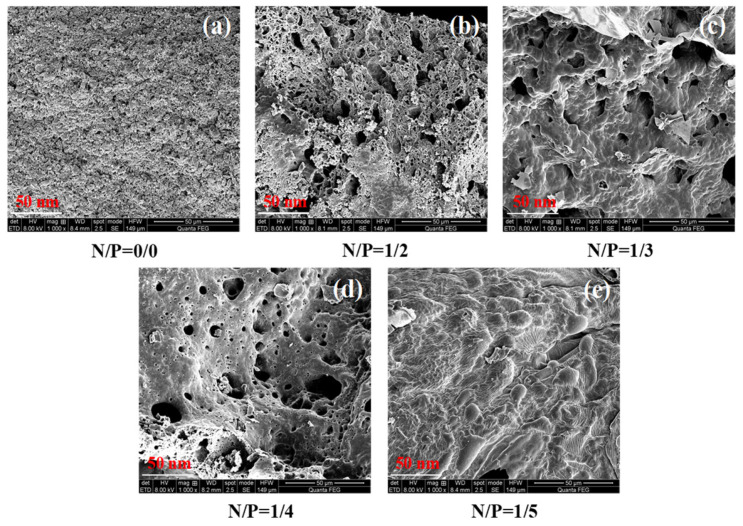
Five groups of gel carbon layer scanning electron microscopy.

A thorough analysis of the carbon layer’s morphology was conducted, revealing the pivotal role of the nitrogen-to-phosphorus ratio in dictating the structural integrity of the carbon layer. The morphology of the combustion residues from gels with different N/P ratios was observed using scanning electron microscopy (SEM), as illustrated in Figure 17. As demonstrated in the accompanying figure, the utilisation of SEM for the precise thickness measurement of the carbon layer cross-sections revealed that the thickness of the carbon layer exhibiting an N/P ratio of 1/4 was found to be 2.5 ± 0.11 mm, signifying a 150% increase in comparison with the control group. A scale bar was included for reference; the surface was dense and free of obvious cracks, whereas the carbon layer in the control group was less than 1 mm thick and exhibited a loose, porous structure. Subsequent observation has indicated that the carbon layer at the optimal ratio (N/P = 1/4) contains a significant number of APTES-modified nanoscale silica sol particles. These particles exhibit a strong binding affinity with the carbon matrix via Si-O-P bonds, thereby significantly enhancing the carbon layer’s high-temperature resistance and mechanical strength.

Distinct gradient changes emerge in the evolution patterns of the pore structure of the carbon layer across different N/P ratios. The control group (N/P = 0/0) exhibits a highly dense residue surface with virtually no pore formation, rendering it incapable of establishing an effective thermal and oxygen barrier. The N/P = 1/2 group begins to exhibit pore structures, though the number of pores is limited and their distribution is uneven. The N/P = 1/4 group demonstrates a significant increase in porosity, forming a rich, uniformly distributed porous network structure that can effectively block heat conduction and oxygen permeation. The N/P = 1/3 group retains porous structural characteristics, but the richness and uniformity of the pores are slightly inferior to those of the N/P = 1/4 group. The N/P = 1/5 group exhibits a substantial reduction in pores, with the structure tending towards densification. This suggests that an excessively high phosphorus ratio disrupts the carbonisation mechanism, creating an imbalance between the rates of gas escape and carbonisation. Consequently, the porous structure could not form effectively, resulting in a decline in flame-retardant performance.

The aforementioned structural evolution patterns suggest that the nitrogen-to-phosphorus ratio plays a key regulatory role in the formation of the porous carbon layer structure. An optimal nitrogen-to-phosphorus ratio facilitates the development of a consistent, uniform porous barrier via a synergistic process involving the formation of gas escape pores and increased carbon matrix strength. Conversely, excessive phosphorus leads to densification of the structure due to its destructive, acid-induced effects, thereby diminishing flame-retardant functionality. In this research system, the optimal nitrogen-to-phosphorus ratio is between N/P = 1/3 and N/P = 1/4, with N/P = 1/4 being closest to the optimum. At this ratio, the carbon layer achieves the best balance of porosity, uniformity and structural integrity.

## 3. Conclusions

This study proposed a novel interface-engineered sodium alginate-based fire-suppressing gel via nitrogen–phosphorus synergistic design and SiO_2_-APTES interface modification. The following conclusions were drawn:

(1) This study presents an interface-engineered, sodium alginate-based nitrogen–phosphorus synergistic flame-retardant gel. Precise control of the N/P molar ratio enabled effective modulation of gelation behavior, network architecture, and rheological properties, with optimal overall performance achieved at N/P = 1/4. The gelation mechanism, governed by combined ionic crosslinking and Si−O−P covalent bonding, offers a new strategy for the structural design of biomass composite gels.
(2) The nitrogen–phosphorus gel system exhibits a synergistic gas−solid biphasic flame-retardant mechanism. The nitrogen source quenches free radicals and provides inert dilution in the gas phase, while the phosphorus source catalyzes efficient charring in the condensed phase. SiO_2_−APTES interfacial bonding further enhances the high-temperature integrity and thermal stability of the char layer, enabling integrated and efficient flame retardancy and smoke suppression.
(3) The distinct optimal ratios for rheology and flame retardancy stem from different governing mechanisms. Rheology depends primarily on crosslinking density and pH, while flame retardancy relies on gas–solid synergistic charring. Nitrogen quenches radicals and dilutes oxygen; phosphorus catalyzes dense char formation; and the APTES-mediated covalent interface stabilizes phosphorus against high-temperature volatilization.
(4) The bio-based gel produced offers a combination of environmental friendliness, strong rheological properties, highly effective smoke suppression and high-temperature resistance. Its potential applications in engineering include preventing spontaneous combustion in coal mines, controlling forest fires and protecting combustible materials from fire, providing a viable solution for developing high-performance, environmentally friendly flame-retardant gels.

## 4. Materials and Methods

### 4.1. Experimental Materials

Sodium alginate (SA, analytical grade AR), silica sol (SiO_2_, nanoparticle size 10~20 nm, PDI < 0.2, 30% aqueous solution), ammonium polyphosphate (APP, polymerization degree n > 1000, analytical grade AR), phytic acid (PA, 50% aqueous solution, analytical grade AR), and 3-aminopropyltriethoxysilane (APTES, analytical grade AR). All materials were procured from Sinopharm Chemical Reagent Co., Ltd. (Shanghai, China) Reagents were used directly without further purification. Deionised water was employed throughout the experiments.

### 4.2. Gel Preparation Process

Initially, the sodium alginate powder must be weighed out and added to deionised water. In order to prepare a uniform, transparent 4 wt% SA solution, it is necessary to stir at 60 °C for 30 min. Concurrently, the original silica sol (30%) should be diluted to 15 wt%, and stirred at room temperature for 10 min to ensure thorough mixing. Subsequently, equal masses (50 g each) of the aforementioned 4 wt% SA solution and 15 wt% SiO_2_ solution were mixed. Subsequently, the stirring was continued at room temperature for a period of 15 min, until a uniform, stable, milky-white mixture was formed, thereby yielding the substrate mixture.

**Figure 18:**
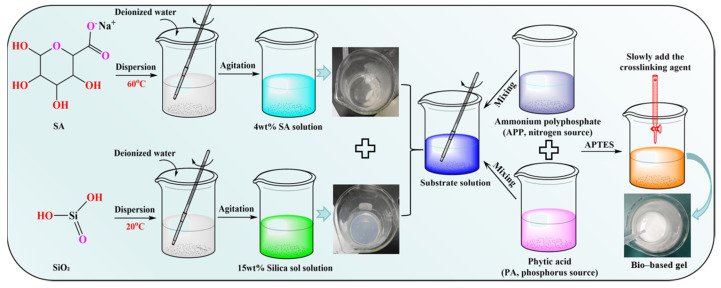
Preparation process of bio-based gel.

An appropriate quantity of APP is weighed and dissolved in deionised water to prepare a 10 wt% APP solution. PA is used directly from its original stock solution (50% aqueous solution). At a temperature of 25 ± 2 °C, the volumes of APP and PA solutions were added sequentially to the base mixture. The mixture was then subjected to homogenisation by magnetic stirring at a rate of 600 r/min for a duration of 5 min. Subsequently, the crosslinking agent APTES was added dropwise at a rate of approximately 1 drop per second, under continuous stirring, until gelation commenced. The stirring process was continued for a duration of two minutes, with the objective of ensuring uniform dispersion of the crosslinking agent. Subsequent to this, the mixture was left to stand in order to observe the formation of gel. The resulting gel samples were then sealed for storage and subsequent performance testing. The bio-based gel preparation process is illustrated in Figure 18. The N/P molar ratio is calculated based on the molar quantities of nitrogen (from APP) and phosphorus (from PA), as shown in Equation (2). The molecular formula of APP is NH_4_PO_3_)n and the molecular weight per repeat unit is 133.04 g/mol. Phytic acid (PA) is C_6_H_18_O_24_P_6_ and the molecular weight is 660.04 g/mol. The base material for the samples was a mixture of 20 g of 4 wt% SA solution and 20 g of 15 wt% SiO_2_ solution. Five sample groups with N/P molar ratios of 0/0, 1/2, 1/3, 1/4, and 1/5 were designed by adjusting the addition amounts of 10 wt% APP solution and 50 wt% PA solution. The precise composition ratios are delineated in Table 2.

(2) $$N/P=\frac{n_{N}}{n_{P}}=\frac{\frac{m_{APP}\cdot w_{APP}}{133.04}}{\frac{m_{PA}\cdot w_{PA}}{660.04}\times 6}$$

### 4.3. Experimental Test Method

#### 4.3.1. Gelation Time Test

The gel formation time was determined by means of the bottle test method. Following the amalgamation of the components in accordance with the ratios stipulated in Table 1, the timing process was initiated without delay. The mixture was stirred until uniform and then left to stand. The flow state of the slurry was observed by means of regular tilting of the beaker, at 30 s intervals. In instances where no substantial flow was detected within five seconds subsequent to tilting the beaker, the timing process was halted, and the recorded time represented the gel formation time. Each sample group underwent three parallel tests, with the mean value taken as the final result.

#### 4.3.2. Viscosity and PH Value Test

The rheological behaviour and viscosity characteristics of the gel were investigated using a shear rheometer (Anton Paar MCR302, Graz, Austria). Tests were conducted at a constant temperature of 25 °C employing a steady-state shear measurement with a flat plate rotor system (25 mm diameter plates, 1 mm gap). The range of shear rates for the experiment was set from 0.1 to 1000 s^−1^, with corresponding variations in shear stress recorded at different rates. The rheometer was calibrated regularly including motor inertia, normal force, and transducer geometry. The gap was accurately verified by the normal force method to ensure 1.0 mm gap distance with no touching errors. All tests followed standard operational protocols.

The pH value was measured using a calibrated digital pH meter (Mettler Toledo FE28, Columbus, OH, USA) with a resolution of ±0.1. The electrode was calibrated with standard buffer solutions (pH 4.01, 6.86, 9.18) before testing. All measurements were performed at 25 ± 2 °C. Freshly prepared gel samples were applied to the pH indicator paper in a uniform manner using a glass rod. Subsequent to a 30 s period of standing, a comparison was made between the paper and a standard colour chart under natural light, and the pH value corresponding to the closest colour block was recorded. Each sample group underwent triplicate parallel testing, with the mean value being taken.

#### 4.3.3. XRD Spectrum Test

The phase composition and microstructure of gels with different N/P ratios were characterized using an X-ray diffractometer (Rigaku SmartLab SE, Tokyo, Japan). The measurements were performed with Cu Kα radiation (λ = 1.5406 Å) at a tube voltage of 40 kV and a tube current of 40 mA. Diffraction patterns were recorded over a 2θ range of 5~90° with a step size of 0.02° and a scanning rate of 5°/min. Prior to analysis, the gel samples were freeze-dried, ground into powder, and compacted on the sample stage.

#### 4.3.4. CCT Test

The flame retardancy of the gel was evaluated using a cone calorimeter (FTT model, Fire Testing Technology, East Grinstead, UK). The combustion behaviour parameters were determined quantitatively based on the oxygen consumption principle under controlled thermal radiation. These parameters included the heat release rate (HRR), the total heat release (THR), the smoke production rate (SPR), and the total smoke production (TSP). Prior to the commencement of the testing procedure, the apparatus was subjected to a preheating process lasting one hour. Zero and full-scale calibrations were conducted on the O_2_, CO, and CO_2_ sensors. A quantity of 20 g of freshly prepared gel sample was weighed and subsequently distributed in a uniform manner across a 100 × 100 mm^2^ aluminium foil container. The heat radiation power was set to 40 kW/m^2^, with a test duration of 10 min. Each sample group underwent triplicate parallel testing, with results averaged.

**Figure 19:**
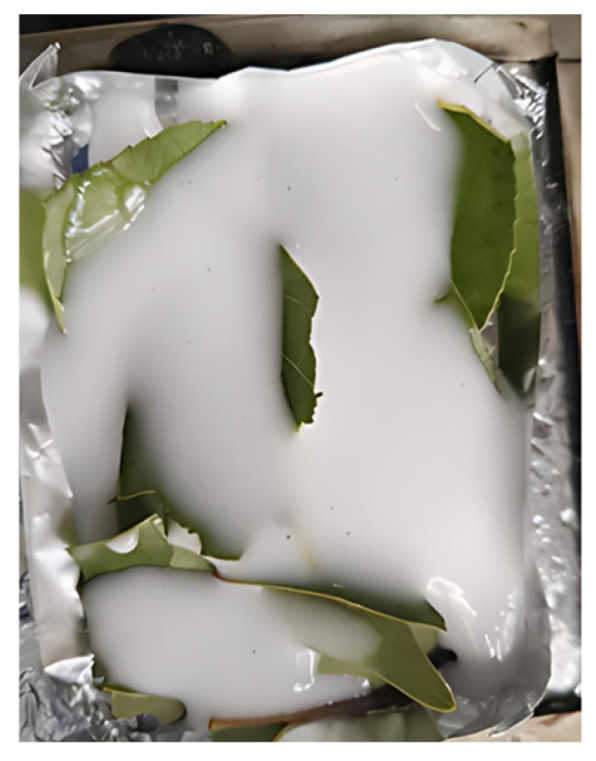
Substrate combustion samples.

To simulate the efficacy of bio-based gels in extinguishing fires in environmental scenarios involving combustible materials, this study selected poplar leaves as a representative combustion substrate. Fresh poplar leaves were collected prior to experimentation, with specimens that were decayed or damaged by insects discarded. The leaves were gently rinsed with deionised water to remove surface impurities and then air-dried in a ventilated, shaded area until they reached a constant weight, thus preserving their natural internal moisture content. The dried leaves were then distributed uniformly across the combustion test area in predetermined quantities to form a standardised combustible substrate layer. The test gel was then uniformly applied to the leaf surface and cone calorimetry testing was conducted under preset conditions. Using poplar leaves as the combustion substrate effectively validated the gel’s potential application in scenarios dominated by combustible materials. The combustion sample of the substrate is illustrated in Figure 19.

#### 4.3.5. FTIR Test

The functional group structure of the gel combustion residue was characterised using a Fourier Transform Infrared Spectrometer (Thermo Fisher Scientific Nicolet iS20, Waltham, MA, USA). The samples were prepared by means of the KBr pressing method. To this end, dried combustion residue was mixed with spectroscopically pure KBr at a mass ratio of 1:100. The mixture was then ground uniformly in an agate mortar and subsequently pressed into transparent pellets using a tablet press. The test conditions comprised a resolution of 4 cm^−1^, 32 scan cycles and a spectral range of 400~4000 cm^−1^.

#### 4.3.6. LRS Test

The carbon structural characteristics of gel combustion residues were analysed using a laser confocal Raman spectrometer (Renishaw inVia model, Wotton-under-Edge, UK). The experimental conditions comprised a laser wavelength of 325 nm, a spectral acquisition range of 300~4000 cm^−1^, continuous scanning mode, and a single scan duration of 2 s. To prevent damage to the sample, the laser spot diameter was adjusted to 4~5 μm and the laser power set to 20 mW. The Raman spectra obtained were then analysed using Labspec 6 software for peak fitting. The intensity ratio of the D peak (1350 cm^−1^) to the G peak (1580 cm^−1^) (ID/IG) was calculated in order to evaluate the degree of graphitisation in the char residue.

#### 4.3.7. TEM-EDS Test

The distribution of elements within the gel was observed using a transmission electron microscope (JEOL JEM-2100F, Tokyo, Japan). The gel sample was subjected to freeze-drying, grinding into a fine powder, dispersion in anhydrous ethanol, and ultrasonic treatment for a period of 10 min. A small volume of the suspension was then transferred onto a carbon film that was supported by a copper grid. Following a drying process at ambient temperature, the specimen was subjected to examination. Energy-dispersive X-ray spectroscopy (EDS) was utilised to conduct an area scanning analysis of the distribution of elements, encompassing C, O, Si, N, and P, at an accelerating voltage of 200 kV.

## Figures and Tables

**Table 1:** ID/IG ratios of char layers with different N/P ratios (mean ± fitting error).

Peer Group	N/P = 0/0	N/P = 1/2	N/P = 1/3	N/P = 1/4	N/P = 1/5
ID/IG	0.792 ± 0.018	0.437 ± 0.012	0.416 ± 0.010	0.267 ± 0.009	0.631 ± 0.015

**Table 2:** Dosage table of different gel components (g).

Peer Group	4 wt% SA Solution	15 wt% SiO_2_ Solution	APP Solution	50 wt% PA Solution	APTES Crosslinking Agent	N/P
1	20	20	0	0	2	0/0
2	20	20	1	0	2	1/2
3	20	20	1	0.8	2	1/3
4	20	20	1	1.8	2	1/4
5	20	20	1	2.8	2	1/5

## Data Availability

The data presented in this study are not publicly available due to confidentiality restrictions associated with ongoing research projects and intellectual property considerations; access to the data may be granted upon reasonable request to the corresponding author, subject to approval by the relevant institutional and funding bodies.

## Nomenclature

APP	Ammonium polyphosphate, −
APTES	γ-Aminopropyltriethoxysilane, −
HRR	Heat release rate, kW/m^2^
ID/IG	The intensity ratio of D peak to G peak, −
*m*	mass of the respective component, g
*w*	mass fraction, −
PDI	Polydispersity Index, −
PA	Phytic acid, −
SA	Sodium alginate, −
SPR	Smoke production rate, m^2^/s
THR	Total heat release, MJ/m^2^
TSP	Total smoke production, m^2^
*Greek letters*	
η	Apparent viscosity, −
γ˙	Shear rate, −
τ	Shear stress, −
